# Low Dose of BPA Induces Liver Injury through Oxidative Stress, Inflammation and Apoptosis in Long–Evans Lactating Rats and Its Perinatal Effect on Female PND6 Offspring

**DOI:** 10.3390/ijms24054585

**Published:** 2023-02-26

**Authors:** Beatriz Linillos-Pradillo, Lisa Rancan, Sergio D. Paredes, Margret Schlumpf, Walter Lichtensteiger, Elena Vara, Jesús Á. F. Tresguerres

**Affiliations:** 1 Department of Biochemistry and Molecular Biology, School of Medicine, Complutense University of Madrid, Avda. Complutense, S/N, 28040 Madrid, Spain; 2 Department of Physiology, School of Medicine, Complutense University of Madrid, Avda. Complutense, S/N, 28040 Madrid, Spain; 3 GREEN Tox and Institute of Veterinary Pharmacology and Toxicology, University of Zürich, Langackerstrasse 49, CH-8057 Zürich, Switzerland

**Keywords:** bisphenol A, oxidative stress, inflammation, apoptosis, liver injury, perinatal offspring

## Abstract

Bisphenol A (BPA) is a phenolic compound used in plastics elaboration for food protection or packaging. BPA-monomers can be released into the food chain, resulting in continuous and ubiquitous low-dose human exposure. This exposure during prenatal development is especially critical and could lead to alterations in ontogeny of tissues increasing the risk of developing diseases in adulthood. The aim was to evaluate whether BPA administration (0.036 mg/kg b.w./day and 3.42 mg/kg b.w./day) to pregnant rats could induce liver injury by generating oxidative stress, inflammation and apoptosis, and whether these effects may be observed in female postnatal day-6 (PND6) offspring. Antioxidant enzymes (CAT, SOD, GR, GPx and GST), glutathione system (GSH/GSSG) and lipid-DNA damage markers (MDA, LPO, NO, 8-OHdG) were measured using colorimetric methods. Inducers of oxidative stress (HO-1d, iNOS, eNOS), inflammation (IL-1β) and apoptosis (AIF, BAX, Bcl-2 and BCL-XL) were measured by qRT-PCR and Western blotting in liver of lactating dams and offspring. Hepatic serum markers and histology were performed. Low dose of BPA caused liver injury in lactating dams and had a perinatal effect in female PND6 offspring by increasing oxidative stress levels, triggering an inflammatory response and apoptosis pathways in the organ responsible for detoxification of this endocrine disruptor.

## 1. Introduction

Bisphenol A [BPA; 2,2-bis (4-hydroxyphenyl)] is a synthetic xenoestrogen compound with a high prevalence in our environment [1,2]. BPA is not hazardous in its polymeric form but is unstable in acidic and basic solutions and when exposed to ultraviolet light. These conditions can convert/transform polymeric BPA into monomeric forms [1]. It is used mainly in the food industry as a monomer in the manufacture of polycarbonate plastics and epoxy resins such as plastic food or beverage containers and in the coating of cans, protecting the contents from direct contact with the metal surface [3,4,5,6,7,8], but also for certain paper products. BPA residues can migrate into the food, beverages or environment due to high temperatures, causing people to inevitably be exposed to BPA in their daily lives [3,6,9,10]. The main source of human exposure is through ingestion [5,9,11], while transdermal absorption and inhalation would be possible through secondary routes of exposure [3,5,9]. BPA can act as an endocrine disruptor showing effects that are similar to those of estrogenic and thyroid hormones. Due to continuous exposure, it can cause health problems in humans, including endocrine, reproductive and metabolic effects, cardiovascular disorders and cancer, so that it has been considered a risk for public health [2,6,9,11]. BPA is absorbed from the small intestine and reaches the liver through the blood, this organ being responsible for its metabolism into its glucuronic acid-conjugated form. Therefore, there is a very real possibility of the presence of a higher concentration and toxicity of this compound in the liver [12]. BPA has also been observed to play a major role in inflammation; as Moon et al. [13] reported, it increases the expression of pro-inflammatory cytokines such as IL-6 and TNF-α. In addition, it also induces an increase in oxidative stress by decreasing antioxidant enzymes [1,3,4,5,7,8] and significantly compromises mitochondrial function [14]. BPA is also able to inhibit cytochrome P450 isoforms in the rat liver [15,16,17]. Other in vivo experimental studies have shown that exposure to BPA can also cause liver disease, including steatosis [18], liver tumors [19] and the metabolic syndrome [20]. In previous studies, BPA has been detected in the human placenta [21], umbilical cord blood [22], amniotic fluid [17,23], fetal liver [24] and breast milk [25] as well as in human serum and urine [26]. Hence, since BPA was found in the previously mentioned tissues, as well as at birth [27], exposure to this compound during prenatal life is probable. However, the effect of BPA on the offspring is still poorly understood. The aim of this study was to evaluate whether BPA administration during pregnancy is able to induce liver damage in lactating rats by affecting the oxidant/antioxidant balance through the induction of oxidative stress, increasing inflammation and triggering apoptosis. Moreover, it was studied whether this effect can also be observed in female offspring at postnatal day 6 (PND6).

## 2. Results

### 2.1. Effects of BPA Exposure on Body Weight, Food Consumption, Number of Pregnant Dams and Mortality Rate in PND6 Offspring and Pup Weight

**Figure 1:**
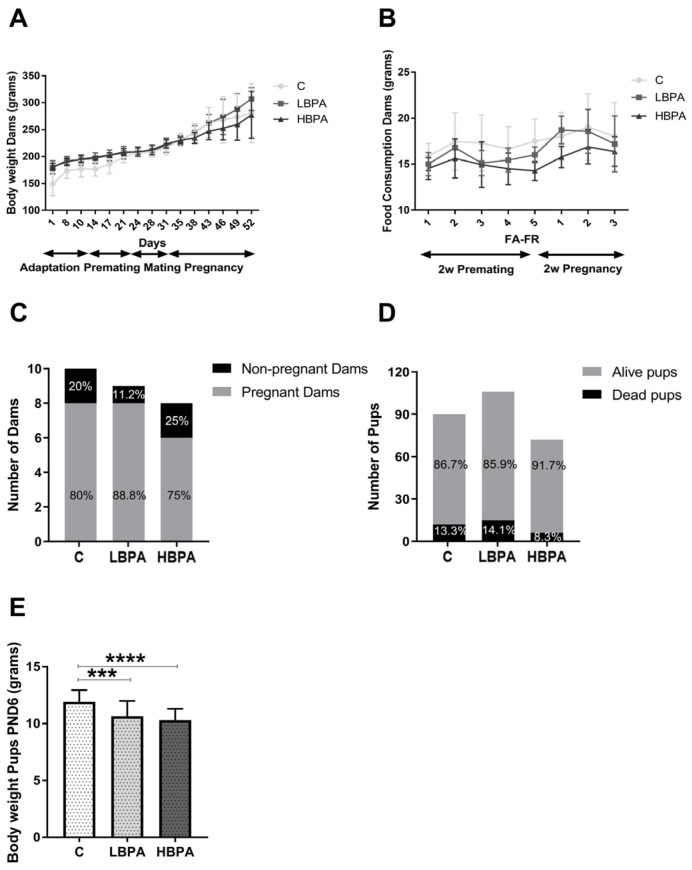
Body weight, food consumption and reproduction data in pregnant dams and neonatal offspring. (**A**) Body weight of dams monitored every 3–4 days; (**B**) food consumption during the second week of premating and the second week of pregnancy; (**C**) number of pregnant and non-pregnant dams; (**D**) percentage of alive and dead offspring after birth and (**E**) body weight of PND6 offspring after birth. Data are represented as mean ± SD. Statistical significance was determined by one-way ANOVA. *** *p* < 0.001, **** *p* < 0.0001.

In this study, clinical observations were made daily and body weight of females was monitored every 3–4 days. Regarding the general appearance, the animals did not show any alteration that could be perceived visually or any unexpected behavior. During the entire experiment it was not necessary to sacrifice any animal for signs of cadence or signs of pain or aggression. Considering all rats from day 24 (before starting mating) at equal weight, an increase in body weight was observed in all females until the maximum weight was reached at the end of pregnancy. No significant differences were observed in the groups of females treated with different doses of BPA in the diet compared to the control group (Figure 1A). There were also no significant differences in food consumption between the control and BPA treatment groups, monitored during the second week of premating and the second week of pregnancy (Figure 1B). Regarding reproduction data, in the case of control females (n = 10), eight female rats were pregnant and two females were not pregnant. In dams treated with BPA, 0.036 mg/kg/b.w./day group (low-dose BPA) (n = 9), it resulted in eight pregnant females and only one non-pregnant female. Regarding dams treated with BPA, 3.42 mg/kg/b.w./day group (high-dose BPA) (n = 8), six females were pregnant and two remained non-pregnant. Therefore, pregnancy was achieved in 22 females from a total of 27 females. The highest percentage of pregnancy was observed in the BPA low dose group (88.8%) followed by the control group (80%) and the lowest percentage of pregnancy was seen in the high-dose BPA group (75%) (Figure 1C). Considering the total number of offspring, the highest number of offspring (106 pups) was obtained in the BPA low dose group followed by the control group (90 pups) and the lowest number of offspring (72 pups) was seen in the high-dose BPA group. However, the mortality rate after birth was higher in the BPA low-dose group (14.1%) compared to the control animals (13.3%) and the BPA high-dose group (8.3%), which had the lowest number of dead pups. Among the living animals, the number of female offspring was 45 in the control group, 46 in the BPA low-dose group and 31 in the high-dose BPA group. All these animals were included in the study. In addition, the offspring of both BPA treatments had lower body weights compared to the control PND6 offspring, while no significant differences were observed between both doses of BPA (Figure 1E). No significant differences were observed between BPA and control groups in the body weight of the females during the entire experiment. In all experimental groups, a constant weight gain was observed, reaching the maximum at the end of pregnancy, as expected (Figure 1A).

### 2.2. Effects of BPA Exposure on Antioxidant Enzyme Activities and Glutathione Concentrations in Dams

**Figure 2:**
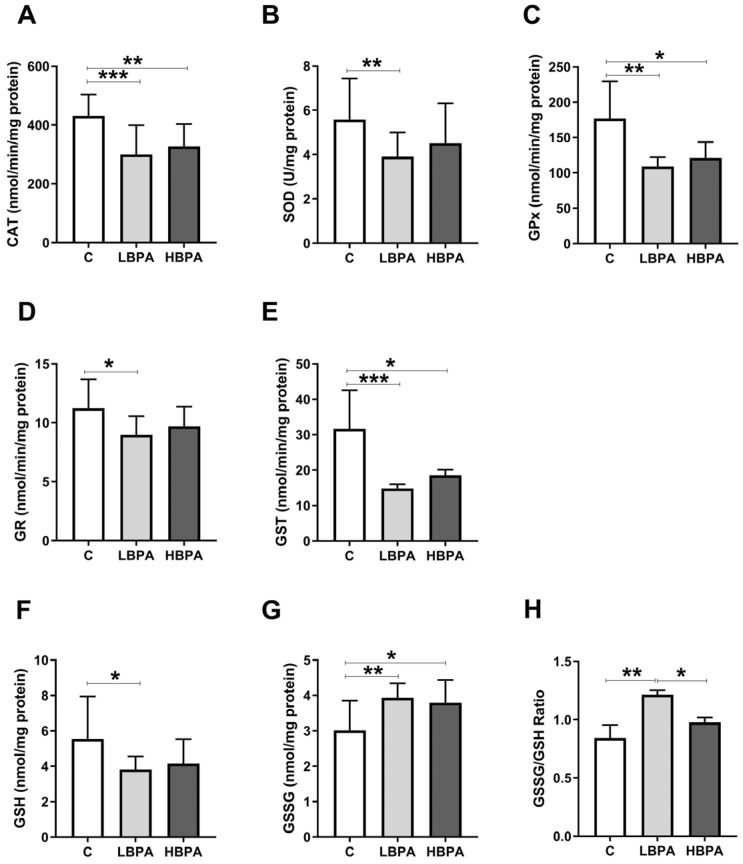
Antioxidant enzyme activities and glutathione concentrations in livers from lactating dams after exposure to low and high doses of BPA. (**A**) Enzymatic activity of catalase (CAT) in nmol/min/mg protein; (**B**) superoxide dismutase (SOD) in U/mg protein; (**C**) glutathione peroxidase (GPx) in nmol/min/mg protein; (**D**) glutathione reductase (GR) in nmol/min/mg protein; (**E**) glutathione S-transferase (GST) in nmol/min/mg protein. (**F**) Concentration of reduced glutathione (GSH) in nmol/mg protein; (**G**) concentration of oxidized glutathione (GSSG) in nmol/mg protein. (**H**) GSSG/GSH ratio. Data represent mean ± SD. n = 8 control dams; n = 8 LBPA dams; n = 6 HBPA dams (two replicates for each sample). Statistical significance was determined by one-way ANOVA. * *p* < 0.05; ** *p* < 0.01; *** *p* < 0.001.

Female rats exposed to low and high doses of BPA were compared in terms of antioxidant enzyme activities and glutathione concentrations; which are endogenous antioxidant defense systems to prevent cellular damage measured in the liver (Figure 2). When lactating females were treated with low-dose BPA, all antioxidant enzyme activities such as catalase (CAT), superoxide dismutase (SOD), glutathione peroxidase (GPx), glutathione reductase (GR) and glutathione S-transferase (GST) were significantly decreased compared to the control group (Figure 2A–E, respectively). In addition, a decrease in reduced glutathione (GSH) concentration (Figure 2F) and an increase in oxidized glutathione (GSSG) concentration (Figure 2G) were observed in low BPA dose-treated dams. When lactating dams were treated with the high dose of BPA, decreased activities of antioxidant enzymes CAT, GPx and GST were observed in comparison to the control group (Figure 2A,C,E, respectively). GSSG concentration was also increased compared to the control (Figure 2G), but no significant differences were observed in the GSH concentration (Figure 2F). The GSSG/GSH ratio, a marker of oxidative stress, was significantly increased in dams exposed to the low dose of BPA compared to the control group and significant differences were also observed between treatment groups, resulting in higher levels of oxidative stress in the low-dose BPA group (Figure 2H).

### 2.3. Effects of BPA Exposure on Oxidative Damage in Dams and Gene Expression Profile of GSH-Related Enzymes

**Figure 3:**
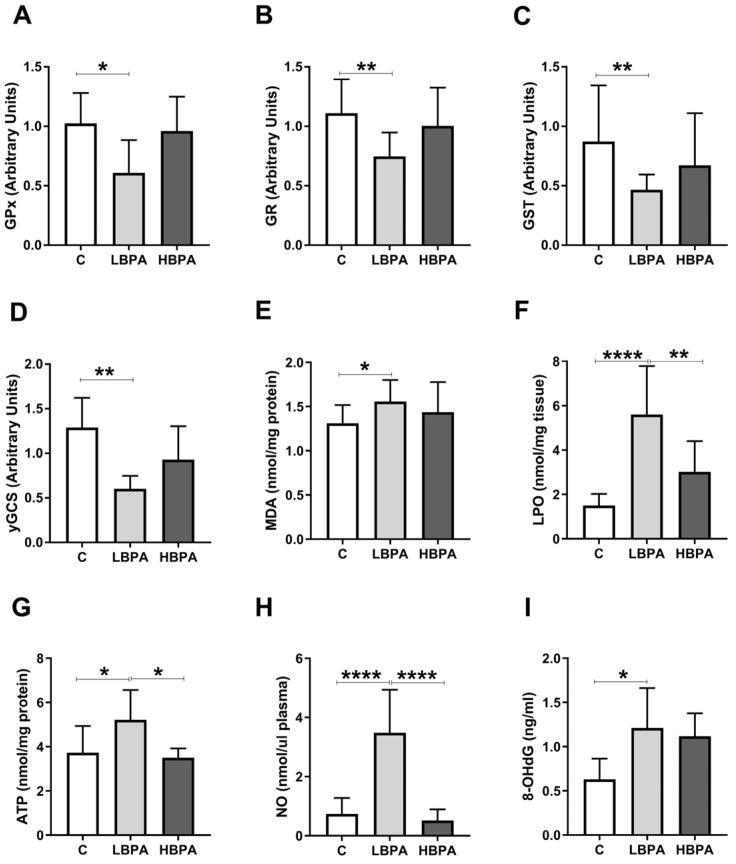
Gene expression profile of GSH-related enzymes and parameters of oxidative damage in livers from lactating dams after exposure to different doses of BPA. (**A**) Glutathione peroxidase (GPx), (**B**) glutathione reductase (GR), (**C**) glutathione S-transferase (GST) and (**D**) γ-glutamylcysteine synthetase (γ-GCS) relative gene expression. (**E**) Malondialdehyde (MDA) content in nmol/mg protein. (**F**) Lipid hydroperoxide (LPO) content in nmol/mg tissue. (**G**) Adenosine triphosphate (ATP) levels in nmol/mg protein. (**H**) Concentration of nitric oxide metabolites (NO) in nmol/μL plasma. (**I**) Concentration of 8-oxo-2′-deoxyguanosine (8-OHdG) in ng/mL. Data represent mean ± SD. For mRNA, n = 8 control dams; n = 8 LBPA dams; n = 6 HBPA dams (three replicates for each gene). For ELISA assay, n = 8 control dams; n = 8 LBPA mg/kg dams; n = 6 HBPA dams (two replicates for each sample). Statistical significance was determined by one-way ANOVA. * *p* < 0.05; ** *p* < 0.01; **** *p* < 0.0001.

Data concerning measurements of oxidative damage and the gene expression of antioxidant enzymes (GPx, GR, GST, γ-glutamylcysteine synthetase (γ-GCS)) were tested in the liver of dams (Figure 3). In the low-dose BPA dams, GPx, GR, GST and γGCS gene expressions were down-regulated versus control dams (Figure 3A–D). Lipid peroxidation is a metabolic process that causes oxidative deterioration of lipids by reactive oxygen species (ROS). This process can degrade lipids within the cell membrane leading to cell damage and eventual cell death. In lactating dams treated with the low dose of BPA, we observed an increase in malondialdehyde (MDA) and lipid hydroperoxide (LPO), two products generated under oxidative stress situations, used to measure oxidative lipid damage (Figure 3E,F, respectively). In dams treated with the low dose of BPA, an increase in 8-oxo-2′-deoxyguanosine (8-OHdG), one of the main DNA oxidation products, used as a biomarker of oxidative DNA damage, was also observed (Figure 3I). Furthermore, an increase in adenosine triphosphate (ATP) energetic levels was observed in the low dose of BPA dams compared to the control group (Figure 3G). Nitric oxide (NO) plays a dual role in oxidative and antioxidant behavior. As an antioxidant, NO protects cells from oxidative stress. However, when produced in excess, it behaves as an important pro-oxidant factor. In this case, an increase in plasma NO metabolites of dams treated with the low dose of BPA was observed (Figure 3H). In addition, when the two BPA doses were compared, significant differences were observed in LPO concentrations, ATP levels and NO metabolites, being significantly lower than the results of the group treated with the high dose of BPA when compared to the low dose (Figure 3F,G,H, respectively).

### 2.4. Effects of BPA Exposure on Oxidative Stress-Inducing Markers in Dams

**Figure 4:**
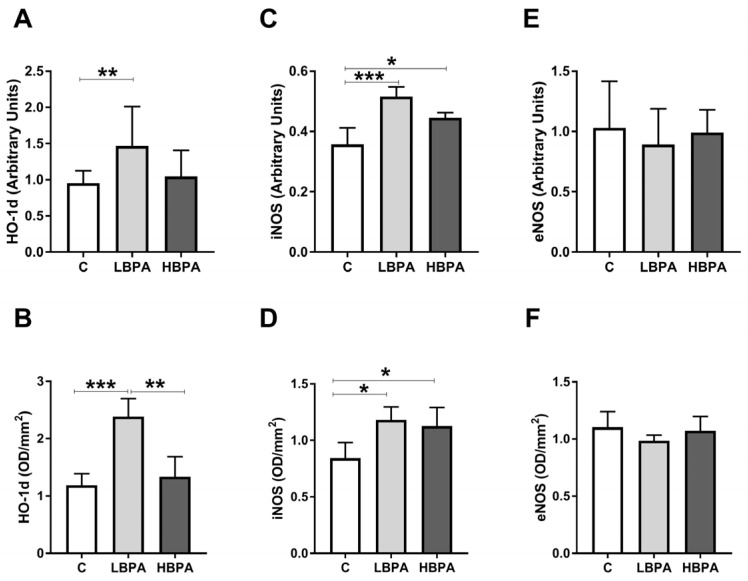
Oxidative stress intermediaries in livers from lactating dams after exposure to different doses of BPA. mRNA and protein expressions of HO-1d (heme oxygenase 1) (**A**,**B**); iNOS (inducible nitric oxide synthase) (**C**,**D**); and eNOS (endothelial nitric oxide synthase) (**E**,**F**). Data represent mean ± SD. For mRNA, n = 8 control dams; n = 8 LBPA dams; n = 6 HBPA dams (three replicates for each gene). For protein, n = 5 rats per experimental group. Data represent mean ± SD. Statistical significance was determined by one-way ANOVA. * *p* < 0.05; ** *p* < 0.01; *** *p* < 0.001.

Results obtained for gene and protein expression of oxidative stress-inducing proteins: Heme oxygenase-1 (HO-1d) and NOS isoforms: Inducible nitric oxide synthase (iNOS) and endothelial nitric oxide synthase (eNOS) in the livers of dams exposed to the two doses of BPA are shown in Figure 4. In dams treated with the low dose of BPA, an up-regulation in gene and protein expressions of HO-1d and iNOS compared to the control group was observed (Figure 4A–D). In dams treated with the high dose of BPA, a significant increase in iNOS gene and protein expressions was observed versus the control dams (Figure 4C,D, respectively). However, no differences in eNOS gene and protein expressions were observed among the groups (Figure 4E,F, respectively). When the two doses of BPA were compared, the only oxidative stress-inducing protein that showed significant differences among groups was HO-1d, which was significantly higher in the low dose of BPA group compared to the high dose one (Figure 4B).

### 2.5. Effects of BPA Exposure in Dams on Inflammatory and Apoptosis Response in the Liver

**Figure 5:**
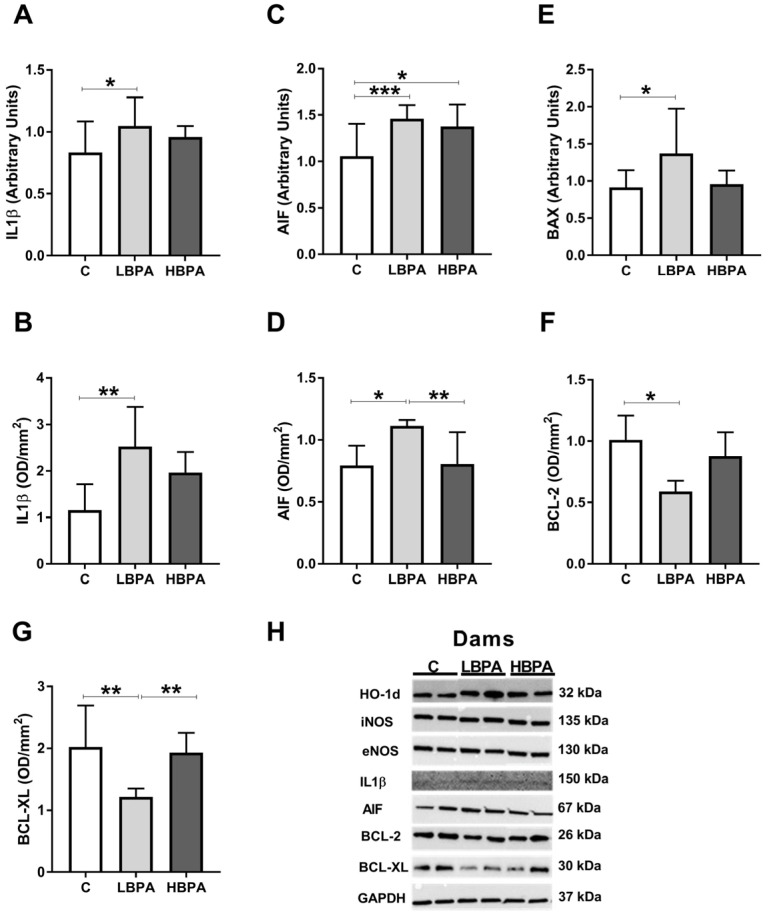
Inflammatory mediator and apoptosis markers in livers from lactating dams after exposure to different doses of BPA. mRNA and protein expressions of IL1β (interleukin-1-β) (**A**,**B**); mRNA and protein expression of AIF (apoptosis-inducing factor) (**C**,**D**). (**E**) mRNA expression of BAX (Bcl-2-associated X protein). (**F**,**G**) Protein expressions of BCL-2 (B-cell lymphoma) and BCL-XL (B-cell lymphoma-extra large). (**H**) Representative images of the Western blot results of the different proteins studied. Data represent mean ± SD. For mRNA, n = 8 control dams; n = 8 LBPA dams; n = 6 HBPA dams (three replicates for each gene). For protein, n = 5 rats per experimental group. Statistical significance was determined by one-way ANOVA. * *p* < 0.05; ** *p* < 0.01; *** *p* < 0.001.

The results of gene and protein expressions of inflammatory markers such as interleukin-1-β (IL1β) and apoptosis markers: Apoptosis-inducing factor (AIF), Bcl-2-associated X protein (BAX), B-cell lymphoma (BCL-2) and B-cell lymphoma-extra large (BCL-XL) are shown in Figure 5. The proinflammatory cytokine IL1β showed a significant increase in gene and protein expressions in dams treated with the low dose of BPA as compared with the control group (Figure 5A,B, respectively). Regarding the proapoptotic molecules, AIF gene and protein expressions were up-regulated in dams treated with the low dose of BPA (Figure 5C,D, respectively) compared with the control group. BAX gene expression was up-regulated in the low dose of BPA treated dams (Figure 5E). In dams treated with high dose of BPA, an increase in AIF gene expression was observed compared to control (Figure 5C). Considering the antiapoptotic molecules, BCL-2 and BCL-XL protein expressions were down-regulated in dams treated with the low dose of BPA (Figure 5F,G, respectively). When the two doses of BPA were compared, a significant difference was found in the protein expression of the proapoptotic molecule AIF, the low dose of BPA group being significantly higher than the high-dose group (Figure 5D). In addition, the protein expression of BCL-XL, an antiapoptotic molecule, was significantly lower in the low dose of BPA group compared to the high dose one (Figure 5G). Representative protein blots for each tested marker are shown in Figure 5H.

### 2.6. Effects of Perinatal Exposure to BPA on Antioxidant Enzyme Activities and Glutathione Concentrations in Liver of Female PND6 Offspring

**Figure 6:**
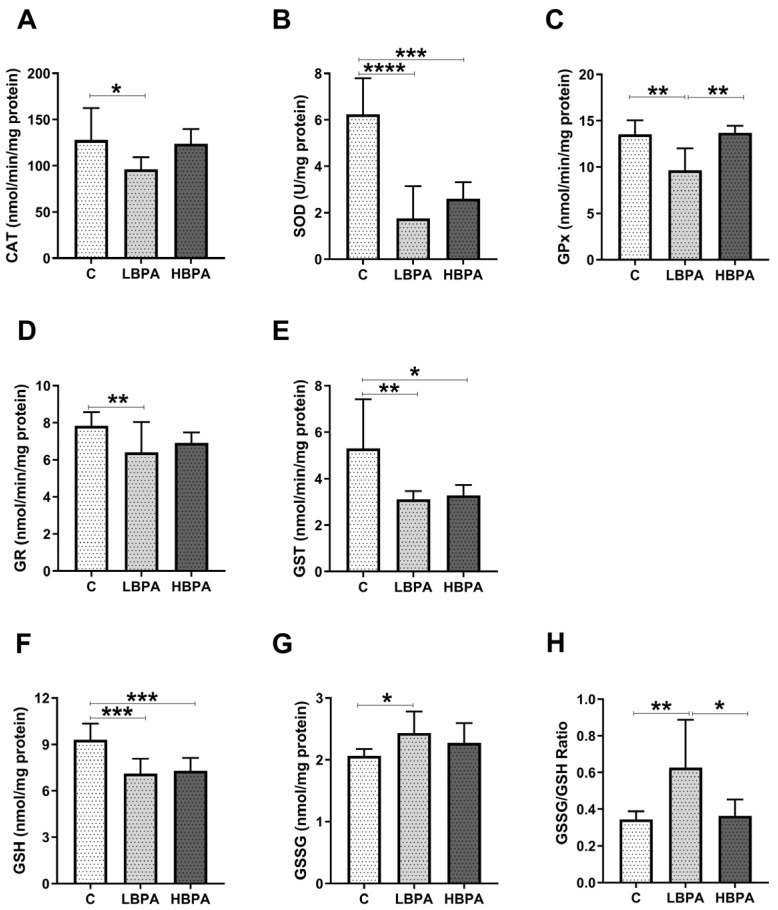
Effects of perinatal exposure to BPA on antioxidant enzyme activities and glutathione concentrations in livers from female PND6 offspring. (**A**) Enzymatic activity of catalase (CAT) in nmol/min/mg protein; (**B**) superoxide dismutase (SOD) in U/mg protein; (**C**) glutathione peroxidase (GPx) in nmol/min/mg protein; (**D**) glutathione reductase (GR) in nmol/min/mg protein; (**E**) glutathione S-transferase (GST) in nmol/min/mg protein. (**F**) Concentration of reduced glutathione (GSH) in nmol/mg protein; (**G**) concentration of oxidized glutathione (GSSG) in ng/mg protein. (**H**) GSSG/GSH ratio. Data represent mean ± SD. n = 12 control PND6 offspring; n = 12 LBPA PND6 offspring; n = 12 HBPA PND6 offspring (two replicates for each sample). Statistical significance was determined by one-way ANOVA. * *p* < 0.05; ** *p* < 0.01; *** *p* < 0.001; **** *p* < 0.0001.

Antioxidant enzyme activities and glutathione concentrations were determined in the livers of female PND6 pups to determine the effect of perinatal exposure to low and high doses of BPA (Figure 6). When PND6 offspring were perinatally exposed to low dose of BPA, all antioxidant enzyme activities (CAT, SOD, GPx, GR and GST) were significantly decreased compared to the control group (Figure 6A–E). In addition, a decrease in reduced glutathione (GSH) concentration (Figure 6F) and an increase in oxidized glutathione (GSSG) concentration (Figure 6G) were observed in the low dose of BPA offspring. These same effects were observed in lactating dams exposed to the low dose of BPA (Figure 2). When PND6 offspring were perinatally exposed to the high dose of BPA, decreased activities of antioxidant enzymes SOD and GST were observed in comparison to the control group (Figure 6B,E, respectively). GSH concentration decreased in comparison to the control group whereas no significant changes were observed in GSSG concentration (Figure 6G). As observed in lactating dams, GSSG/GSH ratio increased in offspring exposed to low dose of BPA as compared to the control group (Figure 6H). When antioxidant enzyme activities and glutathione concentrations were compared between treated groups, a significant increase in GPx was observed in the high dose of BPA group compared to the low dose one (Figure 6C). On the contrary, an imbalance between GSSG and GSH levels was observed in the low dose of BPA group, resulting in a higher ratio as a marker of oxidative stress compared to the high dose one (Figure 6H).

### 2.7. Effects of Perinatal Exposure to BPA on Oxidative Damage and Gene Expression Profile of GSH-Related Enzymes in Liver of PND6 Offspring

**Figure 7:**
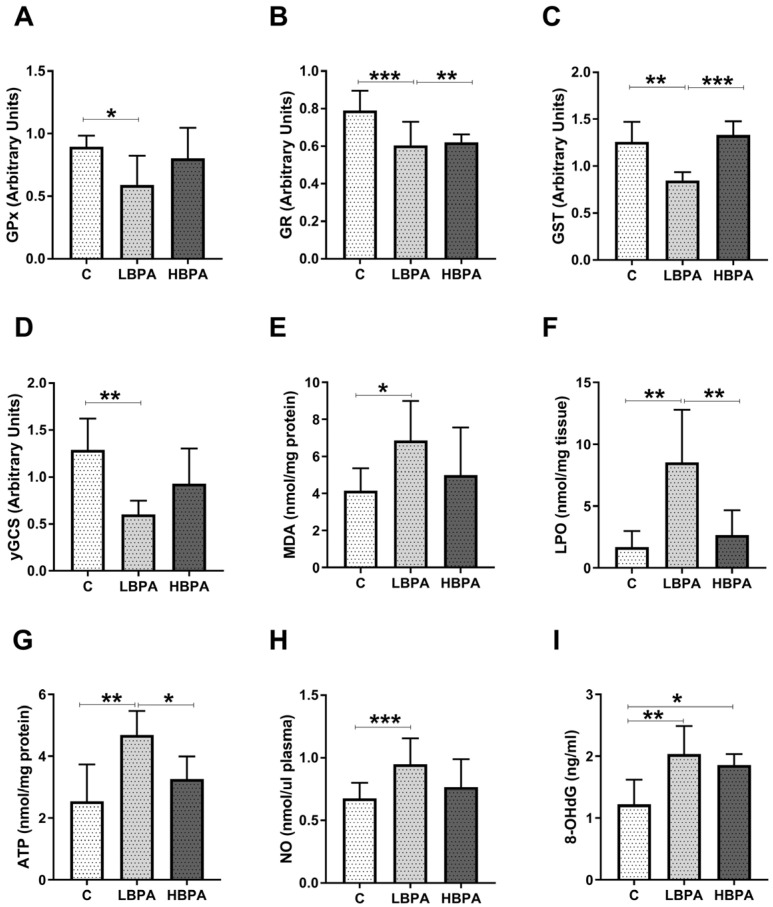
Effects of perinatal exposure to BPA on gene expression profile of GSH-related enzymes and oxidative damage in livers from female PND6 offspring. (**A**) Glutathione peroxidase (GPx); (**B**) glutathione reductase (GR); (**C**) glutathione S-transferase (GST); (**D**) γ-glutamylcysteine synthetase (γ-GCS) relative gene expression. (**E**) Malondialdehyde (MDA) content in nmol/mg protein. (**F**) Lipid hydroperoxide content in nmol/mg tissue. (**G**) Adenosine triphosphate (ATP) levels in nmol/mg protein. (**H**) Concentration of nitric oxide metabolites (NO) in nmol/μL plasma. (**I**) Concentration of 8-oxo-2′-deoxyguanosine (8-OHdG) in ng/mL. Data represent mean ± SD. For ELISA assay, n = 12 control PND6 offspring; n = 12 LBPA PND6 offspring; n = 12 HBPA PND6 offspring (two replicates for each sample). For mRNA, n = 12 control PND6 offspring; n = 12 LBPA PND6 offspring; n = 12 HBPA PND6 offspring (three replicates for each gene). Statistical significance was determined by one-way ANOVA. * *p* < 0.05; ** *p* < 0.01; *** *p* < 0.001.

The transcriptional levels of antioxidant enzymes (GPx, GR, GST, γGCS) and markers of oxidative damage in the liver of female PND6 offspring are shown in Figure 7. GPx, GR, GST and γGCS gene expressions were down-regulated in low-dose-PND6 offspring versus control offspring (Figure 7A–D). In PND6 offspring perinatally exposed to a low dose of BPA, an increase in MDA, LPO and 8-OHdG content compared to the control group was observed (Figure 7E,F,I, respectively). ATP energy levels increased in low-dose-BPA offspring compared to the control group (Figure 7G). These results suggest that perinatal exposure to low doses of BPA increased oxidative damage of lipids and DNA in offspring, as it was observed in dams exposed to low doses of BPA (Figure 3E,F,G,I). Furthermore, an increase in plasma NO metabolites of low-dose-BPA offspring was observed (Figure 7H). In offspring exposed to high doses of BPA, an increase in 8-OHdG was observed, showing oxidative DNA damage (Figure 7I). When the two BPA doses were compared, significant differences were observed in GST gene expression, which was significantly higher in the high-dose-PND6 group compared to the low dose one (Figure 7C). In addition, LPO concentrations and ATP levels showed significant differences between groups, the high-dose-PND6 group being significantly lower compared to the low dose one (Figure 7F,G, respectively). These results are similar to those observed in dams, where significant differences were also observed between treated groups the high dose of BPA group being the one that showed significantly lower levels (Figure 3F,G, respectively).

### 2.8. Effects of Perinatal Exposure to BPA on Oxidative Stress Intermediaries in Liver of PND6 Offspring

**Figure 8:**
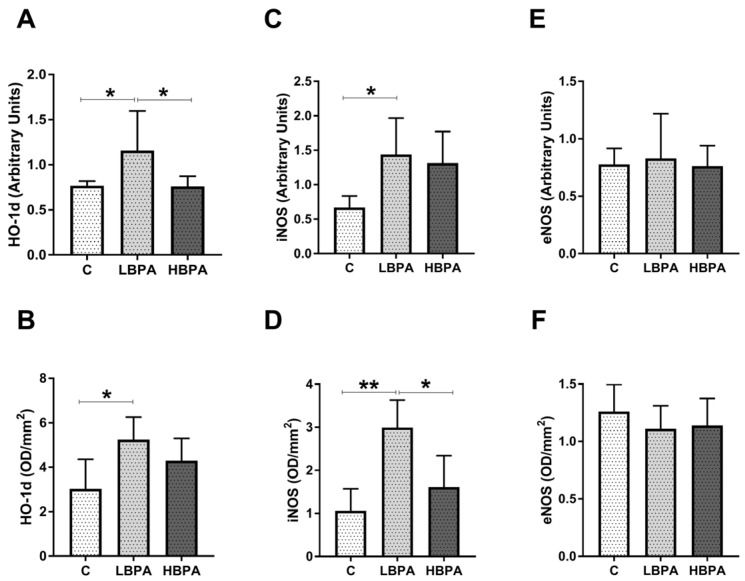
Effects of perinatal exposure to BPA on oxidative stress intermediaries in livers from female PND6 offspring. mRNA and protein expressions of HO-1d (heme oxygenase 1) (**A**,**B**); iNOS (inducible nitric oxide synthase) (**C**,**D**); and eNOS (endothelial nitric oxide synthase) (**E**,**F**). Data represent mean ± SD. For mRNA, n = 12 control PND6 offspring; n = 12 LBPA PND6 offspring; n = 12 HBPA PND6 offspring (three replicates for each gene). For protein, n = 5 rats per experimental group. Statistical significance was determined by one-way ANOVA. * *p* < 0.05; ** *p* < 0.01.

In PND6 offspring exposed to low doses of BPA, an up-regulation in gene and protein expressions of HO-1d and iNOS compared to the control group was observed (Figure 8A–D). Regarding HO-1d gene expression and iNOS protein expression, significant differences were also observed between treatment groups, where the low-dose-PND6 group showed significantly higher expressions than the high dose group (Figure 8A,D, respectively). However, no differences in eNOS gene and protein expressions were observed among groups (Figure 8E,F, respectively); these results were similar to those observed in dams exposed to low doses of BPA (Figure 4).

### 2.9. Effects of Perinatal Exposure to BPA on Inflammatory Mediator and Apoptosis Markers in Liver of PND6 Offspring

**Figure 9:**
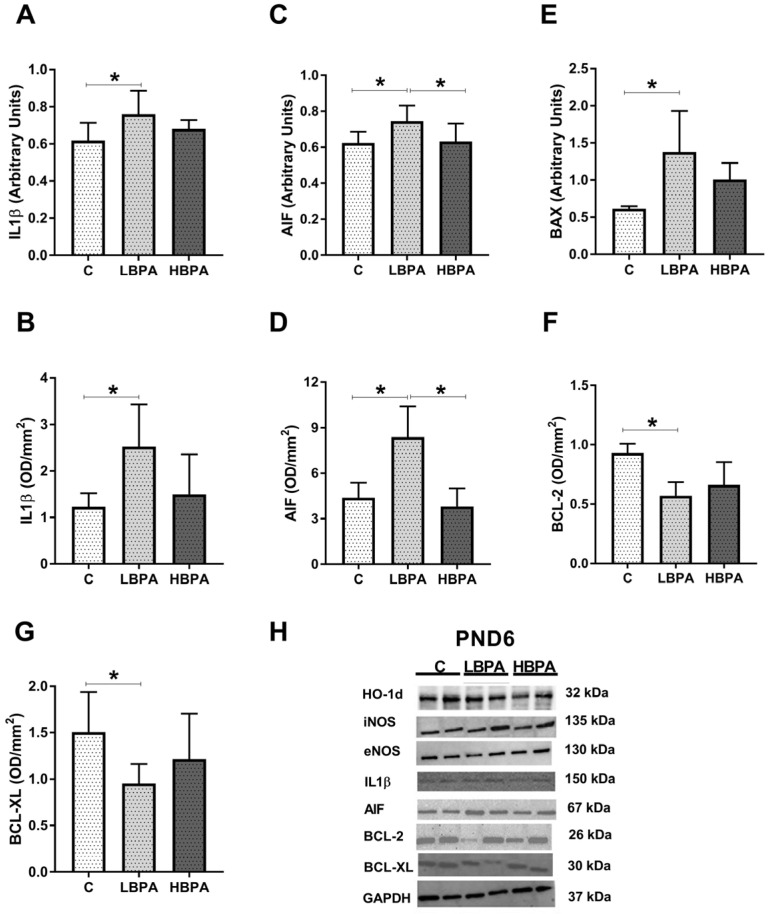
Effects of perinatal exposure to BPA on inflammatory mediator and apoptosis markers in livers from female PND6 offspring. (**A**,**B**) mRNA and protein expressions of IL1β (interleukin-1-β). (**C**,**D**); mRNA and protein expression of AIF (apoptosis-inducing factor). (**E**) mRNA of BAX (Bcl-2-associated X protein). (**F**,**G**) Protein expressions of BCL-2 (B-cell lymphoma) and BCL-XL (B-cell lymphoma-extra large). (**H**) Representative images of the Western blot results of the different proteins studied. Data represent mean ± SD. For mRNA, n = 12 control PND6 offspring; n = 12 LBPA PND6 offspring; n = 12 HBPA PND6 offspring (three replicates for each gene). For protein, n = 5 rats per experimental group. Statistical significance was determined by one-way ANOVA. * *p* < 0.05.

The results of gene and protein expressions of inflammatory markers IL1β and apoptosis markers AIF, BAX, BCL-2 and BCL-XL are shown in Figure 9. The proinflammatory cytokine IL1β showed a significant increase in gene and protein expressions in offspring treated with low doses of BPA as compared to the control group (Figure 9A,B, respectively). Regarding the proapoptotic molecules, AIF gene and protein expressions were up-regulated in offspring exposed to low doses of BPA compared to the control group (Figure 9C,D, respectively). When AIF gene and protein expressions were analyzed between treated groups, significant differences were found. In both gene and protein expressions, the low-dose-PND6 offspring showed significantly higher values than the high-dose group, whose results were not different from the control group (Figure 9C,D, respectively). BAX gene expression was up-regulated in low dose of BPA offspring (Figure 9E). The anti-apoptotic markers BCL-2 and BCL-XL significantly reduced their protein expression in low-dose-BPA offspring versus the control group (Figure 9F,G, respectively). This imbalance between pro-apoptotic and anti-apoptotic family members shown in low-dose-BPA pups was also observed in lactating dams (Figure 5F,G, respectively). Representative protein blots for each tested marker are shown in Figure 9H.

### 2.10. Effect of BPA on Histopathology and Hepatic Serum Markers

**Figure 10:**
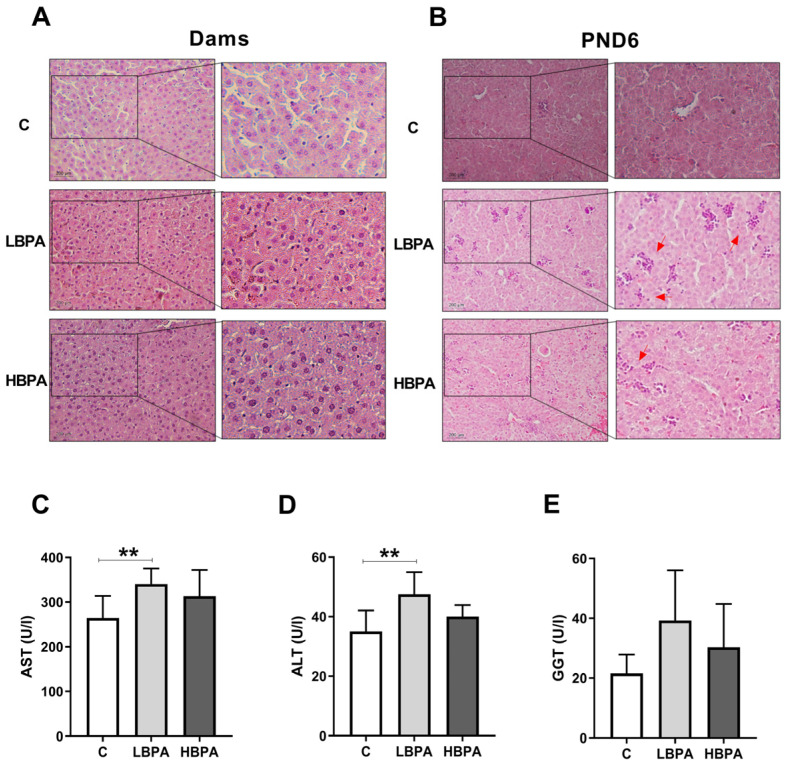
Histology and clinical chemistry of liver functional markers after BPA administration. Representative images of livers from dams (**A**) and female PND6 offspring (**B**) stained with H&E in control and low and high BPA treatment groups (LBPA or HBPA). Scale bars indicate 200 µm (20×) and magnified image of the characteristic tissue section (40×). Red arrows indicate the aggregation of nuclei. (**C**) Aspartate aminotransferase (AST); (**D**) alanine aminotransferase (ALP) and (**E**) gamma glutamyl transpeptidase (GGT) in U/L in serum dams. Data represent mean ± SD. n = 8 control dams; n = 8 LBPA dams; n = 6 HBPA dams. Statistical significance was determined by one-way ANOVA. ** *p* < 0.01.

Hematoxylin and eosin staining was used to analyze the effect of BPA on liver injury; this is shown in Figure 10. In the livers of dams exposed to BPA, no changes were observed in cellular structure compared to control hepatocyte images (Figure 10A). However, histological staining showed that BPA administration increased nucleus aggregation and infiltration of inflammatory cells in PND6 offspring liver tissue compared to control pups. In addition, lower concentration of BPA had a noteworthy impact on liver injury compared to higher doses in PND6 offspring (Figure 10B). Results of hepatic serum marker assessment indicated that dams treated with lose-dose BPA exhibited liver injury manifested by a significant rise in the levels of aspartate aminotransferase (AST) and alanine aminotransferase (ALP) when compared to the control (Figure 10C,D, respectively). The serum levels of gamma glutamyl transpeptidase (GGT) did not show any significant change in animals receiving BPA in comparison with the control group (Figure 10E).

## 3. Discussion

Bisphenol A is one of the most widely used industrial chemicals worldwide. Trasande et al. [28] reported that 90% of the general population has detectable levels of BPA. These BPA levels are 70 times higher in occupationally exposed individuals than in environmentally exposed populations [29]; therefore, BPA exposure is considered an unavoidable and concerning situation. Since BPA exposure occurs mainly through ingestion, in the present study the effects of BPA administered by the oral route at two different concentrations were evaluated: a low dose of 0.036 mg/kg/b.w./day and an almost 100-fold higher dose of 3.42 mg/kg/b.w./day, in the liver of pregnant dams and their perinatal effect in PND6 offspring.

The liver is the main organ responsible for the metabolism of BPA through conjugation by the liver enzyme uridyl diphosphate glucuronyl transferase (UDPGT) to a less toxic compound called bisphenol A-glucuronide (BPAG) [30,31]; this being the main pathway for the detoxification of this xenobiotic. A smaller amount of BPA reacts with sulfate giving rise to BPA-sulfate (BPAS) [32] or can be oxidized to a catechol followed by further transformation to an O-quinone (4,5-bisphenol-O-quinone) [33]. The catechol-O-quinone couple is capable of redox cycling with generation of ROS and oxidative stress [14]. ROS are cytotoxic agents that cause oxidative damage by attacking the cell membrane but also DNA. The liver has an endogenous antioxidant defense system to prevent cellular damage such as antioxidant enzymes and the glutathione system. The activities of antioxidant enzymes CAT, SOD, GPx and GR were decreased in the livers of dams treated with low doses of BPA compared to the control group. There was also a decrease in the enzymatic activities of CAT and GPx in dams treated with high doses of BPA, without any significant difference with the low doses of BPA group. SOD generally dismutates the superoxide anion radical into hydrogen peroxide, which is degraded by CAT using GSH. The reduction in CAT activity may reflect the inability to remove H_2_O_2_ produced after BPA exposure [15,34].

GST protects the cell by conjugating glutathione (GSH) to electrophilic substrates, generating less reactive and more soluble compounds, being a detoxifying enzyme involved in the metabolism of many xenobiotics [35]. Exposure to both doses of BPA showed a significant reduction in GST activity, reflecting an inability to detoxify this compound. Exposure to low BPA dose also reflects lower gene expression levels of the γGCS that catalyzes the first step in glutathione synthesis, resulting in low cellular levels of glutathione. Glutathione provides a first line of defense against ROS as it can scavenge free radicals and reduce H_2_O_2_ formation in the cell. BPA produces several quinols and semiquinone intermediates that can react with glutathione producing glutathione conjugates, which, in turn, increase oxidative stress levels [7]. GPx utilizes reduced glutathione (GSH) to remove peroxides produced by oxidative stress [36]. On the other hand, GR reduces oxidized glutathione (GSSG) back to GSH using NADPH [7].

In the present study, the GSH depletion shown in the dams treated with low BPA dose along with NADPH oxidation and altered redox homeostasis seems to play an important role in the disruption of antioxidant defense, leading to elevated levels of oxidative stress in liver cells. Oxidative stress produces free radicals that can easily react with cell membrane lipids, proteins and nucleic acids, thus initiating a chain of reactions leading to the production of lipid peroxides [37] and DNA damage [38]. In our results, a significant increase in MDA levels was observed with low doses of BPA and an increase in LPO with both doses of BPA in dams, in this case being higher in the low dose of BPA group compared to the high dose one. In turn, exposure to low doses of BPA led to an increase in oxidative damage to DNA as shown by the increased values of 8-OHdG in the livers of pregnant dams.

We observed that exposure to low BPA dose induced liver damage in rats, affecting the oxidant/antioxidant balance and causing liver injury. Our results are in agreement with many others, such as Acaroz et al. [3] who demonstrated decreased SOD and CAT enzymatic activities and GSH levels in Wistar albino rats exposed to BPA at different oral doses (5, 10 and 20 mg/kg). In another study using a dose of 25 mg/kg in rats for 50 days, an increase in MDA levels and a decrease in GSH levels and SOD and CAT activities in kidney, brain and testis tissues was found [39]. Bindhumol et al. [15] also showed a reduction of antioxidant enzymes (SOD, CAT, GR, GPx) in the mitochondrial and microsome-rich fractions of the liver; while H_2_O_2_ and MDA levels increased in Wistar rats treated with BPA doses ranging from 0.2 to 20 µg/kg. The same occurred in the study by Hassan et al. [40] where antioxidant activities were decreased at doses of 50 mg/kg of BPA in rat livers.

To investigate the involvement of BPA in cellular oxidative stress, eNOS, iNOS and HO-1d were tested as mediators of this process. Vascular function mainly depends on the balance between synthesis/degradation of nitric oxide (NO). NO produced by eNOS is a result of a physiological response that plays an important role in mediating many processes such as vasodilation, immunity and neurotransmission. In our results, we observed no difference in eNOS gene and protein expression in both treatment groups. However, elevated plasma NO levels and higher gene and protein expression of iNOS were observed in dams treated with low BPA dose compared with the control group and high BPA dose. An increase in the synthesis of NO produced by iNOS causes vascular dysfunction and its iNOS activation may have some detrimental effects for liver function. NO is a potent oxidant and a nitrating agent capable of attacking and modifying proteins, lipids and DNA, as well as decreasing antioxidant defenses [41].

Regarding heme oxygenase (HO), which participates in the metabolism of the heme group of hemoproteins, two isoforms have been characterized: one inducible (HO-1d) and one constitutive (HO-2d). The inducible isoform, HO-1d, is expressed under various stimuli, such as oxidative stress and cytokines such as TNFα; being a reliable marker of a proinflammatory and prooxidant state [42]. Our results showed an increase in gene and protein expression of HO-1d on low BPA dose treatment in dams. Its induction following increased oxidative stress could act as a cellular defense mechanism to prevent progression of liver fibrosis. Kazemi et al. [1] showed an increase in HO-1d gene expression with a BPA dose-dependent profile in liver cells.

High levels of oxidative stress have been linked to inflammatory processes. In this study, dams treated with low BPA dose increased gene and protein levels of the proinflammatory cytokine (IL-1β). In accordance with our results, Acaroz et al. [3] showed that BPA exposure at 25 mg/kg in male Wistar rats increased the expression of proinflammatory cytokines such as TNF-α, IL-6 and IL-1β and decreased anti-inflammatory/antifibrotic cytokine (IL-10). Elswefy et al. [8] administered 50 mg/kg of BPA to rats orally for eight weeks and reported that its administration significantly increased the serum level of IL-1β and reduced the level of IL-10. This increase in proinflammatory cytokines induced liver inflammation by transporting mononuclear and polymorphonuclear leukocytes to inflamed tissues [43]. In our study, no structural changes of hepatocytes were noticeable yet after BPA administration in the liver of the dams. However, in the histological study by Kazemi et al. [44] it was demonstrated that oral administration of BPA by gavage at low doses induced liver injury in male adult rats.

Liver tissue damage can be assessed by serum liver markers. In our study, a marked increase in AST and ALT was observed with the low BPA dose, indicating tissue damage in the liver. This is consistent with the study by Ijaz et al. [45] where a substantial increase in the levels of alanine aminotransferase (ALT), alkaline phosphatase (ALP) and aspartate aminotransferase (AST) was also observed in BPA-treated rats. This may be because overproduction of ROS damages the structural integrity of liver cells, which is manifested by an increase in hepatic serum markers [46]. However, it is not yet apparent in histological sections because it is at an early stage of involvement after seven weeks of BPA exposure.

Previous studies found that BPA impairs hepatic mitochondrial function by releasing soluble factors into the cytosol [13,47]. This membrane permeabilization may be the initial stage of mitochondrial apoptosis [6]. One of the proapoptotic markers is AIF, which upon release into the cytosol, translocates to the nucleus where it triggers apoptotic pathways. In our study, elevated gene levels of AIF were found in the liver upon exposure to BPA. We also studied the mRNA expression of BAX, another factor that promotes apoptosis, which showed significantly elevated gene expression levels in dams treated with low BPA dose, whereas protein expression of Bcl-2 and BCL-XL, which are anti-apoptotic factors that protect the cell from various cytotoxic alterations, were found to be significantly decreased with the low dose of BPA treatment. Previous studies also showed increased pro-apoptotic protein caspase-3 and reduced anti-apoptotic protein BCL in the liver of male rats [6,8]. BPA weakened hepatocyte mitochondrial function and promoted cell apoptosis in the liver by up-regulating protein levels of Bax, cleaved caspase-3 and cleaved PARP1, while it down-regulated Bcl-2 in the liver using high doses of BPA [48]. Notably, cytochrome c, a key mediator of apoptosis through activation of caspases in the cytosol [49,50,51], was also found to be increased.

Low BPA dose treatment showed elevated ATP levels in pregnant dams compared to the control group. This maintenance of sufficient ATP levels together with the release of pro-apoptotic factors causes liver cells to enter apoptosis [52]. The mechanism of BPA-induced apoptosis probably also involves an alteration in the expression ratio of pro-apoptotic and anti-apoptotic proteins of the BCL-2-associated X family (BAX) and BCL-2 in the outer mitochondrial membrane that modulates the release of proapoptotic factors [53,54]. Therefore, exposure of pregnant dams to low doses of BPA may exert toxic effects on liver cells through the formation of ROS, induction of inflammation and apoptosis.

At high doses of BPA these effects are not as noticeable or significant in many of the parameters studied compared to the control group. This may be because there is a higher level of vulnerability in the liver towards low doses of BPA compared to other organs due to the initial metabolism of BPA by the liver [12,13,55]. BPA is considered a xenoestrogen, but not an estrogen mimic [56] due to its ability to bind to the classical nuclear estrogen receptors (ER) ERα and ERβ [57]; although compared to 17β-estradiol the affinity is about 10,000 times lower for ERα and 1000 times weaker than the affinity for ERβ [58]. It is also able to bind to classical and non-classical membrane estrogen receptors [59], as well as to the G protein-coupled receptor 30 (GPR30) [60], and act through non-genomic pathways [61] and also as an activator of the thyroid hormone and androgen receptors [59,62]. This may explain that BPA, as an endocrine disruptor, such as some hormones, can follow non-monotonic dose–response curves (NMDR), showing more noticeable effects at low doses than at high doses [63]. The endocrine system is configured to respond to very low concentrations of hormones and a maximal biological response can be observed without a high receptor occupancy of this response. This could be due to the fact that response mechanisms become saturated before all receptors are occupied.

This is consistent with a previous study that observed a non-monotonic relationship in pregnant Wistar rats exposed to BPA (50, 250 or 1250 μg/kg) and their offspring after weaning. Only the lowest dose of 50 μg/kg of BPA produced effects such as increased body weight, elevated serum insulin and impaired glucose tolerance in adult pups. However, this study exposed rats to normal or high-fat diets, which could also play a role in the response mechanisms. Rats exposed perinatally to the higher doses showed none of the adverse effects, regardless of diet [20].

Most scientific studies have focused on the effect of high doses of BPA in adults, but the effect of low BPA dose on perinatal exposure seems to be more important to take into consideration [64]. Exposure of pregnant dams to BPA is of concern to the developing fetus since it is able to cross the placenta and enter into cord blood and amniotic fluid. This is in addition to the presence of little or no fetal enzymatic activity at all of UDPGT to biotransform it into inactive BPAG [17]. Furthermore, the enzyme β-glucuronidase is highly active in the placenta and can further contribute to increase fetal exposure to free BPA by hydrolysis of conjugated BPA entering the fetal compartment [20,65]. BPA also binds to the estrogen-related receptor gamma (ERɣ), which is highly expressed in the placenta, facilitating the accumulation of BPA and thus increasing the exposure of the developing fetus to this compound causing potential harmful effects to the offspring at very low and sustained doses.

A recent in vitro study showed that activation of the P2X7 receptor after incubation with BPA has been observed in human placental cells, leading to different pathways involved in producing preeclampsia and preterm delivery, through activation of the NLRP3 inflammasome and apoptosis [66]. Nishikawa et al. [67] showed that the presence of free BPA in the liver of fetal rats could be the result of direct transfer of free BPA into the maternal circulation via the placenta, in addition to the hydrolysis of BPAG in the fetal liver.

In the present study, we observed a decrease in the activity levels of antioxidant enzymes CAT, SOD, GPx, GR and GST in PND6 pups exposed to the low dose of BPA. SOD and GST activities were also decreased in the offspring with the high dose of BPA. GSH was reduced in the offspring exposed to the low dose of BPA, with increased levels of oxidized GSH. In addition to decreased antioxidant enzyme activity, lipid peroxidation-associated damage (increased levels of MDA and LPO) was increased in the liver of offspring exposed to low dose, along with increased DNA oxidation in offspring exposed to both doses of BPA. This is consistent with a study in pregnant mice orally exposed to a dose of 100 ng/g BPA from PND7 to PND21, showing that perinatal BPA exposure could induce oxidative damage and alter normal metabolic profiles in the liver [68].

Lin et al. [69] showed that perinatal BPA exposure causes the development of non-alcoholic fatty liver disease (NAFLD) in the offspring of pregnant Sprague-Dawley rats that had access to water containing 1 or 10 μg/mL BPA from gestational day six (GD6) to PND21. BPA exposure is associated with up-regulation of lipogenic genes, dysregulation of autophagy and activation of the inflammatory response involving PI3K/Akt/mTOR and TLR4/NF-κB pathways. This oxidant/antioxidant imbalance also became noticeable here as gene and protein expression levels of oxidative stress-inducing proteins (HO-1d and iNOS) were increased in the offspring exposed to the low dose of BPA, along with elevated plasma NO levels. Increased proinflammatory cytokine IL-1β and proapoptotic factors AIF and BAX, with the subsequent decrease in antiapoptotic factors BCL-2 and BCL-XL, led to an induction of apoptosis in liver cells in the offspring perinatally exposed to the low dose of BPA. In our study, higher aggregation of nucleus and infiltration of inflammatory cells were observed in the liver of PND6 offspring treated with low dose BPA as compared to the high dose one. Santoro et al. [70] showed that the main histological alteration of the liver was a mild to moderate microvesicular steatosis in BPA-treated rats at 10–17 PND and 45–60 PND. Mild hepatocellular hypertrophy was observed in some BPA-exposed lactating or weaned animals. Furthermore, the expression of inflammatory cytokines, Sirt1, its natural antisense long non-coding RNA (Sirt1-AS LncRNA), and histone deacetylase 1 (Hdac1) were affected in exposed animals. Another study has shown susceptibility to NAFLD in adulthood following mitochondrial dysregulation upon perinatal exposure [20]. Jiang et al. [18] showed that perinatal BPA exposure contributes to the development of hepatic steatosis in male offspring at 3, 15 and 26 weeks when postnatally treated with 40 µg/kg BPA, and that this was mediated by impaired hepatic mitochondrial function.

Therefore, exposure to low levels of endocrine disrupting chemical (EDC) BPA—these levels being easier to achieve in daily life—is of concern since it interferes with many metabolic processes and causes widespread damage to body tissues. The fact that lower levels of BPA are generally more effective than the higher doses is a very remarkable issue as previously described. Moreover, it should also be noted that the timing of BPA exposure may determine the long-term outcome, as earlier exposure points tend to exert a more severe effect [18]. Thus, fetuses and newborns are more sensitive than adults, and chemical exposure during critical developmental stages could cause irreversible long-term consequences [6,17,71]. In our study, similar effects were observed in perinatally exposed offspring as well as in their lactating dams after BPA exposure, being this a critical period influencing ontogenic development of various tissues and also increasing the risk of developing diseases later in adulthood. Further research is critical to understand the extent and effect of prenatal exposure to potentially toxic chemicals including BPA.

Our study is subject to a series of technical limitations. First, since it is part of a bigger European Union’s Horizon 2020 Research and Innovation Programme project (ENDpoiNTs; grant number: 825759), at the time of extraction, livers had to be quickly divided and immediately frozen in liquid nitrogen. Therefore, the liver weight could not be measured. These data could have provided additional information regarding a possible hepatic injury. Another technical limitation regarding the analysis of liver functional enzymes is that this could only be performed with dams’ sera. In the case of PND6 offspring, the collected serum volume was insufficient to perform these chemical determinations. Hence, information about hepatic functionality in PND6 offspring is missing. Furthermore, for experimental design reasons, only female pups were included. It would have been interesting to compare the effects of the BPA administration also in male pups. Finally, an important limitation of the study is that, for technical limitations, BPA level determinations either in plasma or liver biopsies are missing. Furthermore, although special attention was paid to avoid any BPA contamination throughout the entire experiment, an interference of background BPA exposure with low-dose treatment may not be completely excluded.

## 4. Materials and Methods

### 4.1. Animals

Twenty-seven female (eight weeks of age) and twelve male (ten weeks of age) Long–Evans rats (Janvier Labs, Le Genest-Saint-Isle, France) were used in the study. The animals were all housed and maintained in a well-ventilated room at 22 ± 2 °C, with automatic light cycles (12 h light/dark) and all had free access to diet and drinking water ad libitum. Rats were housed in special polypropylene cages (Sodispan Research, Coslada, Madrid, Spain) that were manufactured with the lowest chemical composition of Makrolon, a polycarbonate with bisphenol A. Water bottles were made of glass.

### 4.2. Treatment and Experimental Design

Animals were randomly divided into three groups consisting of: (1) Control (non-treated) group—received chow with a corresponding concentration of corn oil (n = 10 females; n = 4 males); (2) bisphenol A (0.5 mg/kg chow) low-dose group—diet intake of 0.036 mg/kg body weight/day of BPA (n = 9 females; n = 4 males); and (3) bisphenol A (50 mg/kg chow) high-dose group—diet intake of 3.42 mg/kg body weight/day of BPA (n = 8 females; n = 4 males). High dose of BPA was chosen in the range of doses (2.5 mg/kg and 50 mg/kg) that consistently induced impairment learning and memory loss in rodents when administered in the perinatal period. While low dose was 100 times lower than that.

The dose ingested by each rat was calculated based on the food intake data per animal which corresponded to 7.3% of body weight. BPA with purity >99% was purchased from Sigma Aldrich (Argovia, Switzerland) (CAS number 80-05-7; article number: 239658). It was dissolved in ethanol and then corn oil at a ratio of 10% ethanol and 90% corn oil. The chosen chow was purchased from Granovit (Argovia, Switzerland) corresponding to a diet of natural ingredients low in phytoestrogens (rather restricted concentrations so that the estrogenic effects were weak) (Granovit AG, Kaiseraugst, KLIBA NAFAG 3317.PX.L15). Rats were bred in special polypropylene cages (Sodispan Research, Coslada, Madrid, Spain) and glass drinking bottles were used to avoid the presence of substances that could also act as endocrine disruptors. A cylindrical environmental enrichment element was included.

**Figure 11:**
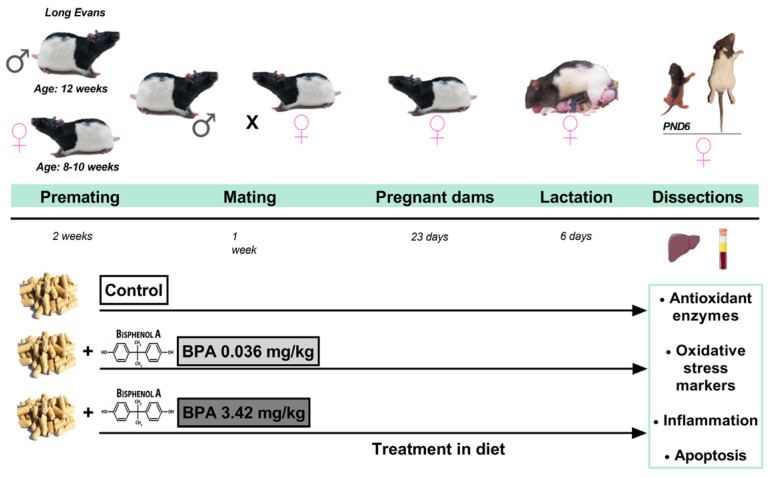
Experimental design. The diet of the parental generation (F0) was different according to the experimental group (control, BPA 0.036 mg/kg and BPA 3.42 mg/kg). During the entire experiment (premating, mating, pregnancy, lactation), the treatment was maintained until the time of dissection in dams and pups to postnatal day 6 (PND6). The influence of lactating dams and perinatal exposure to BPA on livers and its possible mechanism in offspring were studied. Figure created with Prism v7 (GraphPad Software Inc., La Jolla, CA, USA).

During the entire experiment (premating, mating, pregnancy, lactation), the control group cages were kept separate from the BPA-treated groups, to avoid any chance of spreading chow containing BPA. During premating, female and male rats were treated with the diet with their corresponding dose of BPA for two weeks. Control animals received the control diet. Mating phase took place between a male and a female from the same group, after checking that the female was in the estrus phase. The following morning, a check for sperm-positive vaginal smear or sperm-plug was carried out and the process was repeated all mornings for one week. Treatment was maintained during pregnancy. After birth, the lactating dams were kept in individual cages with their offspring and dietary treatment continued until PND6. During the entire period, body weight of females was recorded every 3–4 days and clinical observations were made daily. Furthermore, food consumption (FC) was calculated by weighing the food administered (FA) on the previous day and subtracting weight of food remaining (FR) (FC: FA-FR) during the second week of premating and second week of pregnancy. With 2 rats per cage to avoid isolation stress, we divided the total quantity consumed in the cage by 2 (FC: (FA-FR)/2). In addition to counting the number of pups on the day of birth, the number of dead pups was recorded and the sex ratio and pup weight on PND6 were identified. Lactating dams were sacrificed by decapitation using a guillotine. Female offspring were sacrificed at postnatal day 6 (PND6) by decapitation using scissors. The livers were collected and immediately frozen in liquid nitrogen and stored at −80 °C until analysis. Plasma samples were collected from the lactating dams and stored at −20 °C (Figure 11).

### 4.3. Activities of Antioxidant Enzymes and Glutathione Concentrations

#### 4.3.1. Antioxidant Enzymes

Catalase (CAT), superoxide dismutase (SOD), glutathione peroxidase (GPx), glutathione reductase (GR) and glutathione-S-transferase (GST) activities were measured in the liver homogenate previously lysed with the corresponding buffer and analyzed spectrophotometrically according to the manufacturer’s instructions (Cayman Chemical; Ann Arbor, MI, USA). CAT activity was determined by the reaction with methanol in the presence of an optimal concentration of hydrogen peroxide (H_2_O_2_). The formaldehyde produced was measured spectrophotometrically with 4-amino-3-hydrazino-5-mercapto-1,2,4-triazole as the chromogen at 540 nm. SOD activity was assessed by measuring the dismutation of superoxide radicals generated by xanthine oxidase and hypoxanthine. The standard curve generated using this enzyme provides a means to accurately quantify the activity of all three types of SOD (Cu/Zn, Mn and FeSOD). GPx activity was measured spectrophotometrically; it was coupled to the oxidation of NADPH by GR. A GR assay kit was used to measure activity of this enzyme by quantifying the rate of NADPH oxidation. GST activity was determined spectrophotometrically by measuring formation of the conjugate of reduced glutathione (GSH) and 1-chloro-2,4-dinitrobenzene (CDNB) at 340 nm. Each sample was tested in triplicate. Enzyme activities were normalized according to liver protein content and were expressed as nmol/min/mg of protein except for SOD, which was expressed in U/mg of protein.

#### 4.3.2. Glutathione Concentrations

Liver was homogenized in 50 mM phosphate buffer and 0.1 M EDTA, pH 8. Then, 10 µL of HClO_4_ was added per mL of homogenate and supernatants were used for the quantification of both reduced (GSH) and oxidized (GSSG) glutathione by o-phthalaldehyde (OPT) at pH 12 and pH 8, respectively, resulting in the formation of a fluorescent compound. Fluorescence was measured at 350 nm excitation and 420 nm emission. Results were expressed as nmol of GSSH and GSH per milligram of protein. Moreover, the GSSG/GSH ratio was calculated for each sample.

### 4.4. Oxidative Stress Markers

#### 4.4.1. Lipid Peroxidation Assay

Quantification of lipid peroxidation (LPO) was carried out in liver homogenate according to the manufacturer’s instructions (Cayman Chemical; Ann Arbor, MI, USA). Lipid Hydroperoxide Assay Kit measures the hydroperoxides directly utilizing the redox reactions with ferrous ions. The amount of lipid hydroperoxide was obtained from the linear regression of the standard curve substituting corrected absorbance values for each sample. LPO content was expressed as nmol/mg of tissue. This procedure eliminates any interference caused by hydrogen peroxide or endogenous ferric ions in the sample and provides a more sensitive and reliable assay for lipid peroxidation.

#### 4.4.2. Thiobarbituric Acid Reactive Substances (TBARS) Assay

Lipid peroxidation was also evaluated using a commercial kit (BioVision, Mountain View, CA, USA), which measures the reaction of malondialdehyde (MDA) with thiobarbituric acid (TBA) and the MDA-TBA adduct formation. Samples were resuspended in lysis buffer with the antioxidant butylated hydroxy-toluene (BHT) (0.1 mM) to prevent further formation of MDA during the preparation of the sample or during the heating step. Then, they were centrifuged at 3200× *g* for 30 min. Furthermore, 200 μL of supernatants from each sample were added to 600 μL TBA, and incubated at 95 °C for 60 min. Samples were cooled in ice for 10 min, and 300 μL of n-butanol were added (Sigma-Aldrich, Madrid, Spain) to create an organic phase in which the MDA molecules were to be placed. Samples were centrifuged and 200 μL of upper organic phase were collected and dispensed into a 96-well microplate for spectrophotometric measurement at 532 nm. Results were expressed as nmol TBARS/mg protein.

#### 4.4.3. Adenosine Triphosphate Determination

The adenosine triphosphate (ATP) levels of liver tissue were determined using a colorimetric/fluorometric assay kit (Bio Vision, Milpitas, CA, USA) according to the manufacturer’s instructions. For the assay, 50 mg of liver tissue was used and the ATP content was calculated and expressed as nmol/mg of protein.

#### 4.4.4. Determination of Nitric Oxide Metabolites (NOx)

Levels of nitric oxide metabolites (NO_x_) in plasma samples were measured by the Griess reaction as nitrite ion (NO_2_^−^) concentration after nitrate (NO_3_) reduction to NO_2_^−^. Briefly, after incubation of the plasma with *Escherichia coli* NO_3_ reductase and NADPH^+^ (37 °C, 30 min), 300 µL of Griess reagent (0.5% naphthylenediamine dihydrochloride, 5% sulfonilamide, 25% phosphoric acid (H_3_PO_4_)) (Sigma-Aldrich, Saint Louis, MO, USA) was added. The reaction was performed at 22 °C for 20 min, and the absorbance at 546 nm was measured, using sodium nitrite (NaNO_2_) solution as standard. Results were expressed as nmol/µL of plasma.

#### 4.4.5. DNA Oxidative Damage Measurement

Oxidative DNA damage was assessed by means of an ELISA kit consisting of a competitive assay for the quantitative measurement of 8-hydroxyguanosine (8-OHdG) (Cell Biolabs, Inc., San Diego, CA, USA). The unknown 8-OHG samples or 8-OHdG standards were first added to an 8-OHdG/BSA conjugate preabsorbed microplate. After a brief incubation, an anti-8-OHdG monoclonal antibody was added, followed by an HRP conjugated secondary antibody. The 8-OHdG content in unknown samples was determined by comparison with a predetermined 8-OHG standard curve. Finally, results were expressed as ng/mg DNA.

### 4.5. Determination of Protein Concentration

The protein content of the same samples was evaluated following a bicinchoninic acid protein assay kit protocol (Sigma-Aldrich, Madrid, Spain) or by BCA Assay Pierce (Bio-Rad Laboratories, Hercules, CA, USA) using a BSA standard curve.

### 4.6. RNA Isolation and RT-PCR Quantification

mRNA expression of GPx, GR, GST, γ-glutamylcysteine synthetase (γGCS), heme oxygenase 1 (HO-1d), inducible nitric oxide synthase (iNOS), endothelial nitric oxide synthase (eNOS), interleukin-1-β (IL-1β), apoptosis-inducing factor (AIF) and Bcl-2-associated X protein (BAX) was measured using real time qRT-PCR. RNA was isolated from liver samples according to the method described by Chomczynski [72] using the TRI Reagent Kit (Molecular Research Center, Inc., Cincinnati, OH, USA) following the manufacturer’s protocol. The purity of the RNA was estimated by 1% agarose gel electrophoresis, and RNA concentrations and ratio 260/280 were determined by spectrophotometry BioDrop (Fisher scientific, Waltham, MA, USA). Reverse transcription of 2 mg of RNA for cDNA synthesis was performed using the StaRT Reverse Transcription Kit (AnyGenes, Paris, France). qRT-PCR was performed using a 7500 Fast Real Time PCR System thermal cycler (Applied Biosystems, Cambridge, MA, USA) with the TB Green Ex Taq (Tli RNase H Plus) (Takara Bio Inc., Shiga, Japan) and 300 nM concentrations of specific primers (Table 1). The qPCR amplification cycles were a 95 °C 10 min cycle, followed by 45 cycles at 95 °C for 10 s and at 60 °C for 30 s and finally melting curve analysis, following the recommendations of the manufacturer (95 °C for 10 s, 65 °C for 30 s and 95 °C for 0 s). Amplification of 18S mRNA was used as a loading control for each sample. The gene expression level was analyzed in triplicate for each sample. Relative changes in mRNA expression were calculated using the 2^−∆∆CT^ method [73].

### 4.7. Protein Extraction and Western Blot Analysis

Western blotting was used to measure levels of HO-1d, iNOS, eNOS, IL-1β, AIF, Bcl-2 and B-cell lymphoma-extra large (BCL-XL). Briefly, liver samples, after homogenization with modified RIPA lysis buffer (PBS, Igepal, Sodium deoxycholate (D5670-5G), 10% SDS, PMSF, 0.5 M EDTA and 100 mM EGTA) to which protease inhibitor cocktail (#P-2714) (Sigma-Aldrich, Madrid, Spain), PMSF (#P7626, 1 mM), sodium orthovanadate (#S6506, 2 mM) and sodium pyrophosphate (#S6422, 20 mM) were added, were sonicated and boiled for 10 min at 100 °C in the ratio 1:1 with gel-loading buffer (100 mmol/L TrisHCl (pH 6.8), 4% SDS, 20% glycerol, bromophenol blue 0.1, 200 mmol/L dithiothreitol). Total protein equivalents (25 μg) for each sample were separated by SDS-PAGE by using 10% Mini-PROTEAN TGX Precast acrylamide gels (Bio-Rad Laboratories, Hercules, CA, USA) and were transferred onto a PVDF membrane using the Trans-Blot Turbo Transfer System (Bio-Rad Laboratories, CA, USA). The membrane was immediately placed into blocking buffer containing 5% non-fat milk in 20 mM Tris pH 7.5, 150 mM NaCl and 0.01% Tween-20. The blot was allowed to block at 37 °C for 1 h. The membrane was incubated with a rabbit polyclonal antibody (1:1000) (Table 2) for 12 h at 4 °C, followed by incubation with a goat anti-rabbit IgG secondary antibody (Santa Cruz Biotechnology, Santa Cruz, CA, USA) (1:7000). Protein detection was performed using the Clarity Western ECL Substrate assay kit (Bio-Rad Laboratories, CA, USA) and ECL Plus (Amersham Life Science Inc., Buckinghamshire, UK) by chemiluminescence with the BioRad ChemiDoc MP Imaging System to determine the relative optical densities. Prestained protein markers were used for molecular weight determinations. Housekeeping gene GAPDH was used as loading control (1:5000) (Santa Cruz Biotechnology, Santa Cruz, CA, USA). Proteins were quantified using BioRad Image Lab software v6.1 (Bio-Rad Laboratories, Hercules, CA, USA).

### 4.8. Histological Staining

Liver tissues were washed in 0.9% cold saline and fixed in a 10% formalin buffer solution for the histopathological assessment for 24 h. After fixation, samples were processed for embedding in paraffin. Serial sections (5 µm) were prepared using a rotary microtome Leica RM2125 RTS (Leica Biosystems, Wetzlar, Germany) for hematoxylin and eosin staining (H&E). The sections were stained with 0.1% hematoxylin (Ciba, Basel, Switzerland) for 5 min. Then slides were washed with tap water for 15 min and then a quick wash with hydrochloric alcohol (0.5% HCl in absolute ethanol) to remove excess staining on the sample (differentiation). The acid was neutralized by immersing the sections in tap water for 5 min and a final wash with distilled water. They were immersed in 0.1% eosin (Ciba, Basel, Switzerland) for 5 min. After washing with distilled water, tissue sections were dehydrated using ascending ethanol passages and finishing in xylol for 30 s. Tissue sections were cover slipped. Images were captured with Leica Microscope (Leica Biosystems, Wetzlar, Germany).

### 4.9. Analysis of Hepatic Serum Markers

Evaluation of aspartate aminotransferase (AST), alanine aminotransferase (ALP) and gamma glutamyl transpeptidase (GGT) levels in dams’ sera was determined by a veterinary laboratory (LAV Arturo Soria, Madrid, Spain) using a KONE sequential automatic autoanalyzer (Kemia Científica, Madrid, Spain).

### 4.10. Statistical Analysis

Differences between obtained values (mean ± SD) were assessed by one-way analysis of variance (ANOVA) followed by the Tukey–Kramer multiple comparison test or Bonferroni post-test to compare all pairs of means after testing for normal distribution. A confidence level of 95% (*p* < 0.05) was considered statistically significant. Statistics were calculated using Prism v7 (GraphPad Software Inc., La Jolla, CA, USA).

## 5. Conclusions

Bisphenol A is a molecule capable of producing estrogenic effects and its continued exposure at low doses is unavoidable. It produces adverse effects on the body, including the liver, the main organ in charge of detoxifying the organism. In this study, it was observed that exposure of female Long–Evans rats to low doses of BPA during pregnancy and lactation increased the levels of oxidative stress in the liver, decreasing antioxidant activities and the glutathione system. This loss of homeostasis generated by the excessive accumulation of ROS and NOS caused an increase in inflammation, triggering cellular apoptosis pathways. The effect of perinatal BPA exposure on female offspring was also studied at PND6 and shows similar effects as found in the dams. Although alterations were observed at both doses of BPA, the maximum effect occurred with the low dose of BPA, resulting in an inverse dose–response relationship. We consider that this is especially important since in our everyday life we are constantly exposed to low doses of BPA, with it being present in many commonly used products.

## Figures and Tables

**Table 1:** Primer sequences for quantitative real-time PCR.

Target Gen	Forward (5′-3′)	Reverse (5′-3′)
GPx	CAGTTCGGACATCAGGAGAAT	AGAGCGGGTGAGCCTTCT
GR	GGGCAAAGAAGATTCCAGGTT	GGACGGCTTCATCTTCAGTGA
GST	TTGAGGCACCTGGGTCGCTCTTTAG	GGTTCTGGGACAGCAGGGTCTCAAA
γ-GCS	ATCTGGATGATGCCAACGAGTC	CCTCCATTGGTCGGAACTCTACT
HO-1d	GTCAAGCACAGGGTGACAGA	ATCACCTGCAGCTCCTCAAA
iNOS	CTTTGCCACGGACGAGAC	TCATTGTACTCTGAGGGCTGAC
eNOS	CCAGTGCCCTGCTTCATC	GCAGGGCAAGTTAGGATCAG
IL-1β	TGTGATGAAAGACGGCACAC	CTTCTTCTTTGGGTATTGTTTGG
AIF	AGTCGTTATTGTGGGGTTATCAAC	TTGGTCTTATTTAATAGTCTTGTAGGC
BAX	GTGAGCGGCTGCTTGTCT	GTCCCGAAGTAGGAGAGGA
18S	GGTGCATGGCCGTTCTTA	TCGTTCGTTATCGGAATTAAC

**Table 2:** Source of primary antibodies.

Antibody	Catalog Number	Company
HO-1d	AB1284	Chemicon International, Temecula, CA, USA
iNOS	AB16311	Chemicon International, Temecula, CA, USA
eNOS	AB16301	Chemicon International, Temecula, CA, USA
IL-1β	500-P80	PeproTech EC, Ltd., London, UK
AIF	5318	Cell Signaling Technology, Beverly, MA, USA
BCL-2	2870	Cell Signaling Technology, Beverly, MA, USA
BCL-XL	21061	Signalway antibody, College Park, MD, USA
GAPDH	2118	Cell Signaling Technology, Beverly, MA, USA

## Data Availability

The data that support the findings of this study are available from the corresponding author upon reasonable request.
